# Biomechanics of fencing sport: A scoping review

**DOI:** 10.1371/journal.pone.0171578

**Published:** 2017-02-10

**Authors:** Tony Lin-Wei Chen, Duo Wai-Chi Wong, Yan Wang, Sicong Ren, Fei Yan, Ming Zhang

**Affiliations:** 1 Interdisciplinary Division of Biomedical Engineering, Faculty of Engineering, The Hong Kong Polytechnic University, Hong Kong SAR, China; 2 The Hong Kong Polytechnic University Shenzhen Research Institute, Shenzhen, Guangdong, China; 3 Department of Applied Mechanics, Sichuan University, Chengdu, Sichuan, China; Nanyang Technological University, SINGAPORE

## Abstract

**Objectives:**

The aim of our scoping review was to identify and summarize current evidence on the biomechanics of fencing to inform athlete development and injury prevention.

**Design:**

Scoping review.

**Method:**

Peer-reviewed research was identified from electronic databases using a structured keyword search. Details regarding experimental design, study group characteristics and measured outcomes were extracted from retrieved studies, summarized and information regrouped under themes for analysis. The methodological quality of the evidence was evaluated.

**Results:**

Thirty-seven peer-reviewed studies were retrieved, the majority being observational studies conducted with experienced and elite athletes. The methodological quality of the evidence was “fair” due to the limited scope of research. Male fencers were the prevalent group studied, with the lunge and use of a foil weapon being the principal movement evaluated. Motion capture and pedabarography were the most frequently used data collection techniques.

**Conclusions:**

Elite fencers exhibited sequential coordination of upper and lower limb movements with coherent patterns of muscle activation, compared to novice fencers. These elite features of neuromuscular coordination resulted in higher magnitudes of forward linear velocity of the body center of mass and weapon. Training should focus on explosive power. Sex- and equipment-specific effects could not be evaluated based on available research.

## 1. Introduction

Modern fencing emerged as a competitive sport in Europe and is now a well-recognized Olympic sport, with over 150 member federations [1]. Both the sport and the culture of fencing have progressed significantly over the past decades, with an estimated 22,000 participants in the United States in 2006 [2] and 25,000 in Germany in 2008 [3]. The dressing culture and fighting traditions until the 19^th^ century are likely to have contributed to the promotion of this combat sport [4].

Owing to its unique asymmetry in movement, fencing imposes high physiological demands in terms of neuromuscular coordination, strength and power, and the impact on the musculoskeletal system [5]. As an example, for the basic ‘on-guard’ stance, fencers align their leading foot with their opponent’s position, with the trailing foot placed at an angle to the lead foot for stability [6]. To score against their opponent, fencers must thrust their weapon quickly toward their opponent, which requires an explosive extension of the trailing leg to perform a forceful forward lunge. These quick ‘propulsion’ and ‘dodge’ offense/defense movements further expose fencers to impacts, explosive forces, power absorption, and shear forces of varying magnitude, asymmetrically distributed across the body [7].

Resulted from this dynamic and repetitive movements in fencing matches, fencing injuries were quite prevalent among the athletes. In spite of the rare cases of severe trauma caused by penetration (puncture by broken blades, account for 2.7–3.2%) [2, 8], most of the fencing injuries arise from overuse. In a 5-year survey by the USFA [2], 184 cases of time-loss injuries were recorded for 610 exposures with an overall 30.0% of injury rate. Approximately 52% of all reportable injuries were first or second-degree strains and sprains. Lower limb is most susceptible to injuries. The injury rates were 19.6%, 15.2%, and 13.0% respectively for the knee, thigh, and ankle. These injuries also carry a high risk of chronic morbidity, predominantly achillodynia and patellofemoral pain [9]. Understanding the biomechanics and demands of a sport provides a pathway to injury prevention and safety promotion [10]. An analysis of the biomechanics of a sport can also improve athletes’ skills, tactics and overall performance and competitiveness.

Currently for fencing, biomechanics of performance have been investigated for different movement components of the offensive and defensive manoeuvres and using varying methodologies, which has made interpretation of findings for practice difficult. Therefore, our aim was to perform a scoping review to identify, evaluate and summarize current evidence on the biomechanics of fencing to inform athlete development and injury prevention.

## 2. Methods

### 2.1 Search strategy and study selection

The research was approved by The Human Subject Ethics Sub-committee of The Hong Kong Polytechnic University. The reference number is HSEARS20150814001. As electronic search of five databases was conducted (PubMed, EBSCOhost, Wiley, Web of Science and Google Scholar), using a pre-defined keyword combination (fencing AND (biomechanics OR kinematics OR kinetics OR dynamics OR movements OR performance)) to identify relevant research published in English.

Publication time was not restricted. Nighty-seven articles were identified after duplication removal and screened for eligibility. Inclusion criteria were 1) studies that addressed fencers’ neuromusculoskeletal features and the biomechanics of fencing movements; 2) studies that examined the performance of fencing-specific equipment and training strategy. Studies were excluded if they 1) did not involve human subjects; 2) did not provide numeric results; 3) recruited subjects for sports other than fencing. Literature search was performed on between March 3rd to March 11th, 2016.

During the article screening, titles and abstracts of identified studies were reviewed, independently, by the first two authors to ensure that studies were experimental in nature and addressed the biomechanics of fencing. Papers for retained titles were retrieved for full review to confirm relevance to the aim of our scoping review, as well as to extract required data for analysis: experimental setting and design, characteristics of the study group, sample size, and measured outcomes. Data extraction was done independently by two authors (DWW and YW) of this study. Any inconsistency in the results was solved by group discussion involving a third author (MZ). Based on these summaries of available research evidence, three emergent themes were identified and used to organize our data for analysis: (1) intrinsic, athlete-specific, factors; (2) extrinsic factors; and (3) basic biomechanics.

### 2.2 Quality assessment

Quality of the recruited studies was assessed by two authors (TLC and DWW) using the tool developed by the Effective Public Health Practice Project [11]. Each of following components was rated: selection bias (the likelihood that the selected subjects can represent the target population); study design (the bias resulted from allocation and the independence of exposure and outcomes); confounders (the inter-group imbalance associated with variables that influence intervention or exposure); blinding (concealment of subject allocation and outcome assessment); data collection method (the validity and reliability of outcome measurement); withdrawals and drop-outs; intervention integrity (the percentage of subjects received complete intervention and reports of unintended intervention); analysis appropriate to question (correct statistics and intention-to-treat analysis). A score of ‘strong’, ‘moderate’, and ‘weak’ was assigned to each study according to existing standard [11]. If consensus was not reached, a third author (MZ) made the final decision.

## 3. Results

### 3.1 Search results

**Fig 1:**
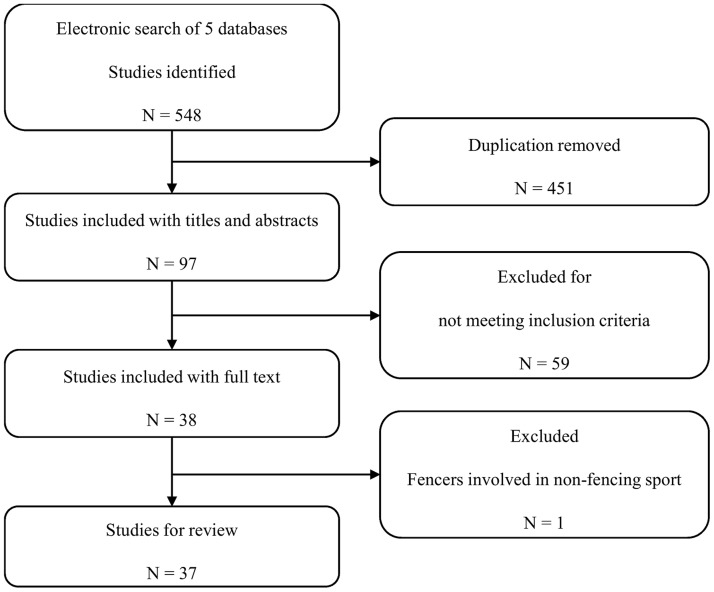
Flow diagram of the search strategy and screening of identified research for inclusion.

**Fig 2:**
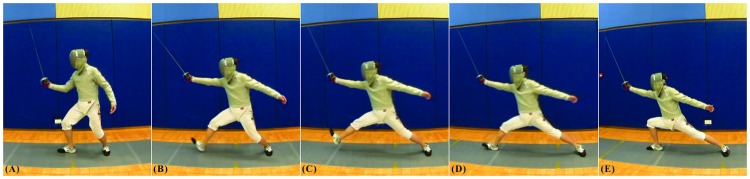
Sequence of movement for lunging using a saber fencer. (A) on-guard position; (B) lifting of the lead leg; (C) forward flying phase of the lead leg and push-off with the trail leg; (D) landing of the lead foot with the armed upper limb in full extension; and (E) final lunge position.

The retrieve results are summarized in Fig 1. We identified 548 studies, with 37 retained for analysis. Among the retained studies, 24 examined the lunge manoeuvre, which was considered to be the core component of fencing (Fig 2). Nine studies did not specify the fencing manoeuvre (Table 1). The biomechanics of the lower limbs was evaluated in 27 studies, and the biomechanics of the upper limbs in 15. The majority of studies were conducted in Europe (70.3%), with three studies conducted in the United States. Expert/elite fencing athletes were the major components of research subjects, which increases the difficulty of enlarging sample size for the recruited studies because top athletes are always rare. All three types of fencing weapons were included in these studies-foils, épées, and sabers. Foils were addressed in 16 studies, épées in 10 and sabers in 6. All studies were lab-based experiments except one performed measurements during competitions (video footage) [12]. Measurements during competitions could provide valuable information of high standard game and athletes. However, the video-based analysis could not quantify fencing biomechanics as accurately as in-lab 3D motion capture technique.

**Table 1:** Basic characteristics of the participants in studies included in our analysis.

Study	Reference number	Location	Subject	Handedness	Age (year)	Mass (kg)	Height (m)	BMI (kg/m^2^)	Weapon	Experience and level
Akpinar et al., 2015	38	United States	4 males 4 females	Right	21.5	N/A	N/A	N/A	N/A	8.8 years on average
Aquili et al., 2013	14	Italy	32 males 25 females	N/A	25.7	71.2	1.79	22.2	Saber	World class
Bottoms et al., 2013	16	United Kingdom	9 males 5 females	Right	26.2	75.6	1.76	24.4	Epee	At least 3 years
Chang et al., 2009	22	Taiwan	8 males	N/A	19.0	65.7	1.73	22.0	Foil	At least 3 years
Cronin et al., 2003	31	New Zealand	21 males	N/A	23.1	76.3	1.76	24.6	N/A	Not fencer athletes
Do and Yiou, 1999	42	France	5 males	Right	25.0	66.0	1.72	22.3	Foil	Not fencer athletes
Frère et al., 2011	17	France	8 males	6 right 2 left	23.0	77.2	1.83	23.1	Epee	10.5 years on average
										National team
Geil, 2002	20	United States	9 males 4 females	N/A	N/A	N/A	N/A	N/A	N/A	3 to 27 years
Gholipour et al., 2008	25	Iran	8 males	Right	22.8	72.2	1.80	22.3	Foil	Novice fencer: college team
										Elite fencer: national team
Greenhalgh et al., 2013	27	United Kingdom	6 males 7 females	N/A	32.4	74.4	1.78	23.5	Foil	At least 3 years
Gresham-Fiegel et al., 2013	32	United States	12 males 13 females	N/A	20.3	N/A	N/A	N/A	N/A	National team
Guilhem et al., 2014	28	France	10 females	N/A	22.2	67.3	1.70	23.3	Saber	National team
Gutierrez-Davila et al., 2013	33	Spain	30 subjects	N/A	35.2	82	1.79	25.6	Epee	National team
Gutierrez-Davila et al., 2013	29	Spain	30 subjects	N/A	29.6	79.8	1.80	24.6	Foil	National team
Hassan and Klauck, 1998	18	Germany	4 females	N/A	16–17	N/A	N/A	N/A	Foil	At least 5 years
Irurtia et al., 2008	26	Spain	16 males 7 females	N/A	18.7	68.9	1.79	21.5	N/A	National team
Kim et al., 2015	36	Korea	9 males	Right	28.2	76.5	1.82	23.1	4 Epee	National team
									5 Saber	
Lin et al., 2010	21	Taiwan	12 males 3 females	Right	19.2	63.2	1.68	22.4	Foil	At least 3 years
Margonato et al, 1994	23	Italy	58 males	N/A	23.0	71.9	1.78	22.7	Foil	11.4 years on average
Morris et al., 2011	15	Canada	1 subject	Right	N/A	N/A	N/A	N/A	Foil	N/A
Mulloy et al., 2015	37	United Kingdom	10 subjects	N/A	22.8	73.6	1.79	23.0	N/A	Novice fencer: at least 1-year experience
										Elite fencer: regional team
Poulis et al., 2009	48	Greece	16 males 14 females	N/A	18.2	62.7	1.73	20.9	N/A	National team
Sinclair and Bottoms, 2015	45	United Kingdom	8 males 8 females	Right	26.1	69.5	1.73	23.2	Epee	Minimum of 3 training sessions per week
Sinclair et al., 2010	7	United Kingdom	19 males	17 right 2 left	25.6	76.8	1.78	24.2	2 Foil	At least 2 years
									16 Epee	
									1 Saber	
Sinclair and Bottoms, 2013	30	United Kingdom	8 males 8 females	Right	26.1	69.5	1.73	23.2	Epee	Minimum of 3 training sessions per week
Sterkowicz-Przybycień, 2009	13	Poland	30 subjects	N/A	23.3	79.1	1.83	23.6	10 Foil	World class
									10 Epee	
									10 Saber	
Steward and Kopetka, 2005	24	United Kingdom	15 subjects	Right	N/A	N/A	N/A	N/A	Epee	N/A
Trautmann et al., 2011	3	Germany	20 males 9 females	N/A	19.3	70.8	1.76	22.9	N/A	National team
Tsolakis et al., 2006	51	Greece	84 males 68 females	N/A	17.0	59.1	1.67	21.2	N/A	Age-based International ranking
Tsolakis et al., 2006	49	Greece	8 males	N/A	12.3	N/A	N/A	N/A	N/A	At least 1 year
Tsolakis et al., 2010	34	Greece	15 males 18 females	N/A	19.9	66.1	1.75	21.6	N/A	National team
Turner et al., 2016	47	United Kingdom	49 males 21 females	N/A	16.8	68.2	1.78	21.5	21 Foil	National team
									30 Epee	
									19 Saber	
Williams and Walmsley, 2000	67	New Zealand	5 males 1 female	N/A	19–26	N/A	N/A	N/A	Foil	Elite fencer: world class
										Novice fencer: local club
Williams and Walmsley, 2000	39	New Zealand	5 males 1 female	N/A	19–26	N/A	N/A	N/A	Foil	Elite fencer: world class
										Novice fencer: local club
Wylde, 2013	12	United Kingdom	9 males	Right	27.4	89.4	1.73	29.9	Foil	Elite fencer: world class
										Novice fencer: not previous fencing experience
Yiou and Do, 2000	40	France	11 males	Right	29.2	72.1	1.71	24.7	Foil	N/A
Yiou and Do, 2001	41	France	11 males	Right	31.0	73.0	1.70	N/A	Foil	N/A

Notes: N/A, not available.

### 3.2 Sample characteristics

Relevant characteristics of the study groups in the 37 retained studies are summarized in Table 1. Male fencers were included to a higher extent than female fencers, overall, and sex-specific effects were not typically addressed. The Body Mass Index (BMI) of fencers was generally within normal limits or slightly lower (23.85kg/m^2^ in fencers VS 24.95kg/m^2^ in the untrained group) because fencers were commonly taller (1.83m VS 1.69m) than the general population [13]. Ethnicity, which would potentially influence anthropometry, was seldom reported, limiting the generalizability of findings. Moreover, two-thirds of the studies enrolled experienced or elite fencers, which further limits the application of findings in developing comprehensive programs for athlete development and injury prevention (in this study, fencing injury refers to those injury types associated with overuse in fencing sports).

### 3.3 Methodological quality

The EPHPP tool assesses many important aspects of the research quality that are critical for studies in public health and injury prevention. The tool is also commonly used by professionals of various topic areas to facilitate their decision-making based on high-quality evidence. In this study, the inter-rater reliability for EPHPP was 0.87 (Cohen’s kappa coefficient), indicating a good agreement between the two reviewers. As showed in Tables 2 and 3, eleven studies (29.7%) were rated as high-quality studies (strong), eight (21.6%) were rated ‘weak’, and the remaining eighteen (48.7%) had ‘moderate’ quality. For most of the recruited studies, they were less scored due to the potential bias present in research design, confounders, and sample selection. Based on the descriptions in the paper, only five studies (13.5%) were randomized controlled trial. However, they were all classified as controlled clinical trial in EPHPP assessment because none of them clarifies the method of randomization in the text. The majority of the studies had case-control design (20 studies, 54.1%) while ten were cross-sectional studies (27.0%). Cross-sectional studies were assigned with ‘weak’ in EPHPP assessment. Some studies recruited less representative samples which had limited control on confounders regarding gender [14, 15], competitiveness [16–18], fencing events [12, 14, 19], and equipment features [20, 21]. Measurement method/collection (e.g. EMG signal processing and motion capture technique) was considered less reliable/not fully elaborated in four studies [13, 22–24]. Statistical analysis was not performed/not introduced in details in five studies [12, 15, 18, 25, 26]. Due to the nature of fencing sports and biomechanical study, outcome assessors could not be blinded to intervention/exposure. Since subjects were generally required to give their best performance in various fencing tasks, we assumed that they were not aware of the research questions for all recruited studies.

**Table 2:** Methodological characteristics of studies included in our analysis.

Study	Reference number	Objective	Moves	Measurement and Equipment	Variable of interest	Results
Akpinar et al., 2015	38	Examined the handedness and performance asymmetries in fencers	N/A	Motion capture (Flock of Birds)	Movement speed	As compared to fencers, non-fencers showed greater inter-limb differences in error making and pointing path deviation under the non-choice condition (p<0.01).
					Movement time	
					Movement accuracy (point error)	
					Movement quality (path deviation from linearity)	Fencers used less right arm to reach middle and left regions under the choice condition (17.0%-23.5% less).
Aquili et al., 2013	14	Time-motion characteristics of saber fencing	Complete bout	Motion capture (Casio & Dartfish)	Time-motion parameters (the type and quantity of actions during the bouts)	There were gender differences in saber data. Males were faster and more frequent in attacking (action/break ratio: 1:6.5 VS 1:5.1, lunge frequency: 23.9/s VS 20.0/s, time of direction change: 65.3s VS 59.7s).
						In both sexes, the percentage of offensive action (49% - 55%) was higher than for defensive action (26% - 31%). The number of lunges was high compared to the number of changes in direction.
Bottoms et al., 2013	16	Identified the kinematic determinants of lunge performance	Lunge	Motion capture (Qualisys)	Kinematics	The average sword velocity was 12.8±3.3m/s, Knee range of motion (30.7°±10.7°) and peak hip flexion of the trailing leg (9.7°±10.9°), and peak hip flexion of the leading leg (102.0°±13.0°) were significant predictors of sword velocity (R^2^ = 0.14–0.36, p<0.01).
					Regression analysis for lunge performance	
Chang et al., 2009	22	Determine appropriate foil handle shape which could reduce the load on grip force	Quarte sixte	EMG measurement (Biometrics)	Muscle activity	The defensive position has no significant effects on muscle activity (p = 0.39). Activity of the adductor pollicis and the extensor carpi radialis (average value = 0.16±0.02) was significantly lower when using the Poistol-Viscounti in comparison to other types of handles.
Cronin et al., 2003	31	Identified the strength qualities predictive of lunge performance	Lunge	Squat performance (Fitness Works)	Squat strength and velocity	The average lunge velocity was 1.62±0.21m/s (concentric velocity), The best three predictors of lunge velocity (R^2^ = 0.85, p<0.05) were time to peak squat force (0.48±0.07s), leg length (83.9±5.2cm), and flexibility (171.0±12.5cm).
					Explosive strength	
				Lunge test (Unimeasure)	Regression analysis for lunge performance	When lunge velocity was normalized by body mass, the three best predictors (R^2^ = 0.87, p<0.05) were time to peak squat force, mean squat power (364.0±96.8W) and relative squat strength (1.65±0.32kg/body mass).
Do and Yiou, 1999	42	Examine the effects of anticipatory postural adjustments on fencing speed	Touche	Force plate (Unspecified)	Displacement of the foot	In lunge + touche condition, when touche was initiated (onset of anterior deltoid) before the postural adjustment of lunge (200ms prior to foot off), touche speed was comparable to that in the isolated touche condition.
				Accelerometer (Entran)	Acceleration of the foil	
			Lunge	EMG measurement (Unspecified)	Muscle activity	When touche was initiated during postural adjustment, touche speed dropped. The average touche speed was significantly lower when executed at the time of foot off (2.19±0.52m/s VS 2.54±0.44m/s, p<0.01) than that in the isolated touche condition.
Frère et al., 2011	17	Classify fencers based on kinematics and muscular activation pattern	Fleche	Motion capture (Vicon)	Kinematics	Experienced and elite fencers did not differ significantly in their anthropometries.
				EMG measurement (Noraxon)	Muscle activity	Fencers were firstly sorted into two groups based on the timing of maximal elbow extension (MEE, early group: 0.20±0.06s, late group: 0.47±0.03s).
						Further EMG-based classification was performed to the two groups. The results showed that early MEE group exhibited higher deltoid intensity (91±18%) than late MEE group (36±13%) in attacking (p<0.05). Spherical classification confirmed that muscular activity was different based on the strategies used in the two groups.
Geil, 2002	20	Effects of different footwear on plantar pressure	Advance	Motion capture (Peak Performance)	Kinematics	The court shoes significantly reduced plantar pressure by 15.37–26.38% as compared to the fencing shoes in all fencing movements.
			Lunge	Plantar pressure (Pedar)	Plantar pressure	Pressures were consistently higher at the front foot. The major pressured regions are the front heel and back medial forefoot (average pressure normalized by body mass: 0.0611–0.0862N/kg∙cm^2^).
			Fleche			The court shoes altered fencers’ kinematics by increasing the range of motion of the weapon hand. The consistency of repeated movement and lunge velocity also reduced in course shoes condition.
Gholipour et al., 2008	25	Compared the kinematics of fencers at different levels	Lunge	Motion capture (Kinemetrix)	Kinematics	Elite fencers had a higher mean lunge length (1.17±0.17m VS 1.02±0.10m), larger late-phase knee extension (51±9° VS 18±8°), and shorter time gap in hand/foot motion (0.07±0.05s VS 0.13±0.15s) in comparison to the novice fencers.
Greenhalgh et al., 2013	27	Effects of sports surface on impact shock during a fencing movement	Lunge	Accelerometer (Biometrics)	Impact shock	Significantly larger impact shock magnitude (F = 17.07, p<0.001) was identified during a lunge on the concrete-based surface (14.9±8.5g) compared with the wooden-based surface (range: 11.1–12.0g). Use of a ‘piste’ had no significant effect on the overall impact shock magnitude (p = 0.38–0.69).
Gresham-Fiegel et al., 2013	32	Effects of trail leg displacement angle on lunge performance	Lunge	Power measurement (TENDO Weightlifting Analyzer)	Lunge power and velocity	For all fencers, their natural trail leg displacement angles were 68° to 100°, 60% of them had a forward deviation, 12% had a perpendicular stance, and 28% had a backward deviation.
						A perpendicular placement (90°) of the feet produced the greatest average power (411.1±97.8W) and velocity (0.59±0.11m/s) during lunging, while forward deviation (45°) produced the lowest values (336.8±70.2W in power, 0.49±0.09m/s in velocity).
Guilhem et al., 2014	28	Investigated the coordination of the leg muscles in fencing execution	Advance lunge	Dynamometer (CMV)	Muscle strength and activity	Concentric contraction tests showed that peak torque produced by hip extensors (221.1±64.0N∙m) and knee extensors (173.4±33.9 N∙m) were significantly greater in the front leg than the rear leg. The front ankle dorsiflexor torque was 20% stronger than the rear leg on the whole range of motion.
				EMG measurement (Zerowire)	Lunge displacement	
				Force plate (Kistler)		E The fencers reached their peak velocity (2.6±0.9m/s) at the early phase 4 of lunge, while peak acceleration (6.5±0.9m/s), force (469.6±77.4N), and power (1051.8±231.5W) occurred in the middle of phase 3.
						Knee extensors of the trailing leg were mainly activated (25.3%-35.1% more than the front leg) during propulsive phases, and less activated than that of the front leg (10.4% more than the trailing leg) during the braking phase.
						Hip extensors of the leading leg were mainly activated (54.1% more than the trailing leg) during the final braking phase.
						Hip and knee extensors and ankle plantarflexors were earlier activated in the trailing leg, while ankle dorsiflexors were earlier activated in the front leg.
Gutierrez-Davila et al., 2013	33	Effects of target change on fencing performance	Lunge	Motion capture (Vicon)	Kinematics	A change in target location significantly increased the reaction time (by 28±32ms), movement time (by 69±50ms), the time used in acceleration (by 43±49ms), and errors made (by 18±19%) during lunge, while it also decreased the attacking velocity (by 0.33±0.35m/s) and action time in front foot (by 0.083±0.023s).
				Force plate (IBV)	Kinetics	
					Time-motion parameters	
Gutierrez-Davila et al., 2013	29	Examined the differences between elite and medium fencers in response to changed lunge target	Lunge	Motion capture (Vicon)	Kinematics	Elite fencers generated higher flight time (36±37ms VS -2±12ms), late-phase horizontal foot velocity (4.56±0.75m/s VS 3.59±0.30m/s), sword velocity (2.55±0.42m/s VS 1.88±0.48m/s), and lunge length (1.40±0.15m VS 1.13±0.13m) as compared to the medium-level fencers.
				Force plate (IBV)	Kinetics	
					Time-motion parameters	Elite fencers made fewer errors (31±17% VS 43±12%) and maintained better arm-foot timing sequence in responses to target change.
Hassan and Klauck, 1998	18	Evaluate the fencing lunge movement based on quantitative analysis	Lunge	Motion capture (SELSPOT II)	Kinematics	The maximal horizontal foil velocity was 3.40–3.91m/s, horizontal foil velocity at hit time was 2.96–3.56m/s, and maximal horizontal hip velocity was 2.28–2.33m/s across four subjects.
Irurtia et al., 2008	26	Assessed the anthropometry and limb asymmetry in Spanish junior fencers	N/A	Anthropometric assessment	Anthropometries	Male fencers showed significantly (p = 0.01–0.05) larger forearm and thigh girths, as well as higher thigh muscle cross-sectional area (236±26 vs. 212±19cm^2^) on the armed side than the Spanish reference population, while females fencers did not exhibit the advantages.
						No inter-limb significant differences were identified in both genders.
Kim et al., 2015	36	Effects of a specific training program in improving muscle imbalance	N/A	Motion capture (Motion Analysis Corporation)	Dispersion of center of mass and center of pressure	Fences showed significant improvement in mediolateral sway of the non-dominant leg during one-leg standing (8.55±4.46cm/fl VS 7.95±1.52cm/fl), mediolateral sway during deep squats (14.76 ±7.18cm/fl VS 9.95±2.54cm/fl) and the balance scale after (3.14±1.72 VS 1.81±0.92) training.
				Force plate (Kistler)	Balance score	
Lin et al., 2010	21	Evaluated the workload of the wrist muscles for different foil handle types	Quarte sixte	EMG measurement (Biometrics)	Muscle activity	The Viscounti-type handle elicited the most equal load distribution for all muscle groups in comparison to other handle types. However, Adductor pollicis and extensor carpi radialis were more activated in Viscounti-type handle condition (p = 0.011–0.017) and may be more vulnerable to fatigue.
						Handle angles for 21° and 24° increased risks of muscle fatigue, Grip strength was highest (8.66–9.52 (unknown unit)) at the two handle angles (p = 0.029).
Margonato et al, 1994	23	Investigate the bilateral differences in forearm muscle trophism and force	N/A	Anthropometric measurement	Dynamometer (Lafayette Instruments)	Fencers exhibited significant differences in cross-sectional area (51.7±8.2cm^2^ VS 45.8±7.8cm^2^) and isometric force (502±126N VS 449±115N) of the forearm between the dominate and non-dominate side (p<0.001).
						Fencers and the control groups were not significantly different on non-dominate side regarding muscular force and trophism.
						The absolute gains in muscular force and trophism of the dominate side were greater in fencers than the control group (5.9cm^2^ VS 2.0cm^2^, 53N VS 15N).
Morris et al., 2011	15	Investigated the characteristics of two fencing movements	Lunge	Motion capture (Vicon)	Kinematics	During the lunge, the ankle plantarflexors and knee extensors of the trailing leg contributed significantly to the attack. On the other hand, ankle plantarflexors and extensors of the hip and knee of both limbs contributed significantly to the progression of fleche. (Results are displayed by graphs only).
			Fleche	Force plate (Unspecified)	Kinetics	
Mulloy et al., 2015	37	Determined the kinematic chain in lunge	Lunge	Motion capture (Motion Analysis Corporation)	Kinematics	Expert fencers exhibited greater peak sword velocity (3.21±0.22m/s VS 2.63±0.29m/s), lunge distance (1.12±0.07 leg length VS 0.83±0.15 leg length), and peak ankle extension velocity (564±132°/s VS 273±184°/s).
						The sequential motion of the hip-knee-ankle sequential is more tightly coupled in elite than in non-elite fencers, allowing elite fencers to achieve greater ankle extension and forward sword velocity. (sequence identified by graphic comparison).
Poulis et al., 2009	48	Examined the asymmetry of muscle strength in fencers	N/A	Anthropometric assessment	Anthropometries	Fencers had greater knee extension torque (112.3–221.4Nm VS 111.7–210.4Nm), flexion torque (66.9–119.7Nm VS 62.3–112.4Nm), and flexor/extensor peak torque ratio (F = 3.04–3.79, p = 0.01–0.03) compared to the non-fencer group, in regardless of the differences in angular velocity (30°/s, 60°/s, and 240°/s).
				Strength test (Cybex)	Isokinetic strength	The differences in peak torque between the dominant and non-dominant legs were not significant in both groups.
Sinclair and Bottoms, 2015	45	Determined sex-differences in joint loading during lunge	Lunge	Motion capture (C-Motion)	Kinematics	Female fencers had significantly greater peak knee extension moment (2.05±0.22Nm∙kg VS 1.72±0.25Nm∙kg), patellofemoral joint contact force (2.90±0.58BW VS 2.18±0.43BW), and contact force loading rate (22.12±5.74BW/s VS 14.14±6.36BW/s) in comparison to male fencers.
				Force plate (Kistler)	Kinetics	
					Joint loading	
Sinclair et al., 2010	7	Effects of footwear on shock attenuating	Lunge	Accelerometer (Biometrics)	Impact shock	Traditional fencing shoes significantly increased the magnitude of peak impact shock in comparison to sports shoes that had shock absorbing qualities (p<0.01).
Sinclair and Bottoms, 2013	30	Investigated sex-differences in fencing kinematics and kinetics	Lunge	Motion capture (C-Motion)	Kinematics	FWhen variables were normalized by body weight., there were no significant inter-gender differences in both kinetics and kinematics except that, female fencers had significantly greater peak hip (42.79±12.42° VS 51.64±10.25°) and knee abduction angles (1.91±6.44° VS -8.99±4.91°) in comparison to male fencers.
				Force plate (Kistler)	Kinetics	
Sterkowicz-Przybycień, 2009	13	Body composition and somatotype of male fencers	N/A	Anthropometric assessment	Anthropometries	Fencers were characterized by higher mesomorphy and lower ectomorphy (p<0.05) compared to untrained males. Fencers' somatotypes differed from that of the untrained (3.3–4.8–2.3 vs. 3.7–4.3–3.1).
						Fencers using sabers were relatively heavier (84.4kg VS 74.9–77.9kg) and had higher mesomorphy (3.4–5.4–1.8 VS 3.6–4.9–2.5 and 2.9–4.2–2.8) than fencers using two other types of weapons.
Steward and Kopetka, 2005	24	Kinematic determinants of lunge speed	Lunge	Motion capture (Peak Motus)	Kinematics	Lunge velocity was significantly correlated to the time-to-peak angular velocity of the trailing knee (p = 0.022) and leading elbow (p = 0.047).
					Regression analysis for lunge performance	
Trautmann et al., 2011	3	Determined the foot loading characteristics of three fencing movements	Lunge	Plantar pressure (Pedar)	Plantar pressure	For the leading leg, the heel was predominately loaded during lunge (peak pressure: 551.8±113.9kPa, contact time: 705.4±166.9ms) while its hallux was more loaded during retreating movements (peak pressure: 341.0±122.4kPa, contact time: 205.0±43.0ms).
			Advance		Time-force parameters	For the trailing leg, the forefoot was generally loaded across the three different fencing movements (peak pressure: 170.2–352.7kPa, contact time: 191.8–682.2ms).
			Retreat			
Tsolakis et al., 2006	51	Investigated the anthropometric profile of young fencers	N/A	Anthropometric assessment	Anthropometries	There were generally no significant differences between male and female fencers in all age group in terms of anthropometric measurements.
						The mean somatotype of male fencers was 3.1–2.6–3.2 as compared to 3.8–14.8–3.3 in females. Female fencers were mainly situated in the ectomorph region.
						Cross-sectional area of the arms was higher in males compared to females and higher on the dominant side compared to the non-dominant side.
Tsolakis et al., 2006	49	Effects of a conditioning program to peripubertal fencers	N/A	Anthropometric assessment	Anthropometries	Increases in anthropometries, hormone level, and handgrip strength were detected in both groups. However, differences between fencers and the inactive children were not significant (p>0.05).
				Blood sampling	Hormones concentrations	
				Not fencing specific physical test (Psion XP)	Muscle strength	
					Physical performance	
Tsolakis et al., 2010	34	Investigated selected correlates of fencing performance	Lunge	Anthropometric assessment	Anthropometries	Lunge time was best predicted (R^2^ = 0.42, p = 0.001) by drop jump performance (30.5±8.58cm) and thigh cross-sectional area (205.3±38.50cm^2^). When lunge time was normalized by body mass, only performance on the arm-driven counter-movement jump (37.7±9.26cm) was predictive of lunge time (R^2^ = 0.71, p<0.001).
				Not fencing specific physical test (Psion XP)	Physical performance	
				Lunge test (Polifermo Radio Light)	Regression analysis for lunge performance	
Turner et al., 2016	47	Determined physical characteristics that underpinned lunge performance	Lunge	Anthropometric assessment	Anthropometries	Standing broad jump (177.7±0.32cm) was the strongest predictor of lunge velocity (3.35±0.70m/s, R^2^ = 0.507, p<0.001) and change of direction speed (5.45±0.65m/s, R^2^ = 0.425, p<0.001).
				Not fencing specific physical test (Optijump)	Physical performance	
				Lunge test (Casio)	Regression analysis for lunge performance	
Williams and Walmsley, 2000	67	Compare response profile between novice and elite fencers under several levels of target choice	Lunge	EMG measurement (Medicotest)	EMG signals timing	The elite fencers showed superiority over the novice fencers in reaction time (32-33ms less), total response time (19-23ms less), onset of activation in anterior deltoid (37-45ms less) and front rectus femoris (52-55ms less) in regardless of the changed target conditions (F = 10.29–34.46, p<0.05).
				Timing record	Timing parameters	Increased target number slightly elongated reaction time and delayed the onset of muscle activation for all fencers. However, the differences were not significant in both elite and novice groups.
						Elite fencers made fewer errors (11 VS 70) in hitting target than the novice fencers (X^2^ = 12.18, p = 0.002).
						For both groups, the within-subject correlation was 75% (all variables of interest), indicating inter-trial consistency of movement pattern.
Williams and Walmsley, 2000	39	Compare response timing and muscle coordination between different fencers under target changing condition	Lunge	EMG measurement (Medicotest)	EMG signals timing	Elite fencers showed shorter reaction time (333±128ms, 40% of total response time VS 613±62ms, 66% of total response time) and total response time (808±53ms VS 934±34ms) in response to changed target.
				Timing record	Timing parameters	Elite fencers exhibited faster activation of selected muscle groups (178-378ms VS 301-617ms) in comparison to the novice fencers.
				Lunge distance measurement	Lunge distance	Elite fencers exhibited more coherent muscle synergy and consistent patterns of muscle coordination (rear knee extensor-front shoulder extensor-front knee extensor-front knee flexor).
Wylde, 2013	12	Time-motion analysis of foil fencing	Complete bout	Time-motion analysis (Sportstec)	Movement timing	High-intensity movements had a mean duration of 0.7s and accounted for 6.2% of the total bout time in elite women foil fencing.
					Movement duration	The work: recovery ratio of female’s foil (15-touch) was 1:1.1, which was similar to that of men’s epee (1:1), men’s foil (1:3), and men’s epee (8:10).
						For the 5-touch and team bouts the work: recovery ratio was 1:1, indicating an increased duration of moderate- and high-intensity movements.
Yiou and Do, 2000	40	Examine the differences between singular and combined training strategy of fencing performance	Touche	Force plate (Unspecified)	Foot pressure	There were no significant differences in body acceleration and peak velocity between the elite and novice fencers when touche and lunge were executed separately.
				Accelerometer (Entran)	Acceleration of body center	
			Lunge	EMG measurement (Unspecified)	Muscle activity	Elite fencers exhibited higher foil velocity (2.90±0.30m/s VS 2.66±0.29m/s) and postural velocity (0.41±0.20m/s VS 0.05±0.09m/s) in the sequential touché + lunge condition compared to the isolated touché condition, while no significant differences emerged for novice fencers.
Yiou and Do, 2001	41	Examined the effects of “refractory period” on fencing movement	Touche	Force plate (Unspecified)	Displacement of the foot	There were no significant differences in postural velocity, speed performance, speed of focal movement, onset of anterior deltoid, and time of target hit between the elite and novice fencers in isolated touche condition.
				Accelerometer (Entran)	Acceleration of the foil	
			Lunge	EMG measurement (Unspecified)	Muscle activity	In lunge + touche condition, when the signal of touche was initiated more than 300ms prior to foot-off, there were no significant differences between groups. When touche was initiated within 200ms prior to foot-off or at the time of postural adjustment, speed performance and speed of focal movement were significantly higher in elite fencers.
						Maximum speed of touché was higher in elite fencers than in novice

Notes: N/A, not available.

**Table 3:** EPHPP score for the included studies.

Study	Research design	Number of subjects	EPHPP Score
Akpinar et al., 2015	Case-control	8	1
Aquili et al., 2013	Case-control	57	2
Bottoms et al., 2013	Cross-section	14	3
Chang et al., 2009	RCT	8	2
Cronin et al., 2003	Cross-section	21	2
Do and Yiou, 1999	Case-control	5	2
Frère et al., 2011	Case-control	8	2
Geil, 2002	RCT	13	2
Gholipour et al., 2008	Case-control	8	3
Greenhalgh et al., 2013	RCT	13	1
Gresham-Fiegel et al., 2013	Case-control	25	1
Guilhem et al., 2014	Case-control	10	1
Gutierrez-Davila et al., 2013	Cross-section	30	2
Gutierrez-Davila et al., 2013	Case-control	30	2
Hassan and Klauck, 1998	Cross-section	4	3
Irurtia et al., 2008	Cross-section	23	3
Kim et al., 2015	Cohort	9	2
Lin et al., 2010	RCT	15	3
Margonato et al, 1994	Case-control	58	2
Morris et al., 2011	Cross-section	1	3
Mulloy et al., 2015	Case-control	10	2
Poulis et al., 2009	Case-control	30	1
Sinclair and Bottoms, 2015	Case-control	16	1
Sinclair et al., 2010	RCT	19	2
Sinclair and Bottoms, 2013	Case-control	16	1
Sterkowicz-Przybycień, 2009	Case-control	30	2
Steward and Kopetka, 2005	Cross-section	15	3
Trautmann et al., 2011	Mixed design	29	1
Tsolakis et al., 2006	Case-control	152	2
Tsolakis et al., 2006	Controlled Clinical Trial	8	1
Tsolakis et al., 2010	Cross-section	33	2
Turner et al., 2016	Cross-section	70	2
Williams and Walmsley, 2000	Case-control	6	2
Williams and Walmsley, 2000	Case-control	6	2
Wylde, 2013	Cross-section	9	3
Yiou and Do, 2000	Case-control	11	1
Yiou and Do, 2001	Case-control	11	1

Notes: RCT, randomized controlled trial; N/A, not available.

In addition to EPHPP score, the main limitation across the studies was the small sample size of study groups, with 14 studies including ≤10 participants. Sample size estimation, based on feasibility studies, was performed in only three studies [7, 27, 28]. Although significance level of 0.05, using two-tailed tests, was the most prevalent cut off for statistical analysis, the cut-off was not clarified in some studies [20, 22]. The small size of study groups increases the risk of violating the assumption of normal distribution required for parametric statistics. Yet, normality for analysis of variance and t-tests was verified in only six studies [7, 17, 27–30]. Therefore, the statistical methodology of studies included in our analysis was only “fair”, with none of the studies reporting the statistical power of their results. Effect size, using Cohen’s d-statistic (range in included studies, 0.29–2.26) and the eta-square statistic (range in included studies, 0.09–0.59) were reported in only six studies. The reliability of measured outcomes was evaluated by computing the interclass correlation coefficient (range in included studies, (0.570–0.988) in four studies [31–34].

### 3.4 Measurement methods

Techniques used for measurements are summarized in Table 2. Motion capture was used in 15 studies and force platform in 10 studies respectively, with electromyography measurement used in nine studies and accelerometers in five studies. However, there was noticeable variation in equipment and signal processing/analysis used across studies, even within a specific measurement technique. As an example, both infrared-based retroreflective marker [15, 17, 18, 29, 33, 35–37] and electromagnetic sensor tracking systems [38] were used in motion capture analysis. In terms of the processing of electromyography data, some studies used the root mean square of the signal [21, 28] while others normalized the signal to the magnitude of a maximal voluntary contraction [17, 22, 39–42].

## 4. Discussion

### 4.1 Intrinsic, athlete-specific, factors

#### 4.1.1 Sex-specific differences

As commonly reported for other sports, sex-specific differences in the kinematics of fencing were identified [35, 43, 44]. Specifically, females exhibited greater hip adduction, knee abduction/adduction, ankle eversion and patellofemoral contact forces of the leading leg during lunging movements [30, 45]. Some researchers have proposed that sex-specific differences in anthropometrics, neuromuscular functions and muscle strength may account for these differences in the kinematics of the leading leg [35, 43, 44]. Overall, the prevalence of injury was higher in female fencers (29%-44%, averaged: 36%) than in males (22%-32%, averaged: 27%) [2]. However, further evidence is required to characterize sex-specific differences based on high-quality evidence.

#### 4.1.2 Anthropometry and muscle strength

The assessment of anthropometrics and strength is an important component for establishing functional profiles of fencers [46]. Performance of the lunge, considered to be the core component of fencing, was evaluated using stepping time or velocity, counter-movement jump strength and dynamometric tests [28, 47], with some studies evaluating the value of squat jump tests for predicting lunge performance [31, 34]. Despite differences in measurement, there is a consensus that greater lower limb strength and explosive power generated higher lunge velocity and quicker fencing moves [48, 49]. Ballistic training is therefore recommended to increase the rate of muscle force generation [50, 51], or a combination of resistance and ballistic training to optimize the explosive, power and endurance requirements of fencing [51, 52]. Turner et al. reported that most training programs were customized to a fencer’s ability, sex and age by coaches, based on their experience or anecdotal knowledge rather than on evidence. Turner et al. [53] advocated the need to develop evidence-based training protocols which would consider a fencer’s biomechanics, as well as physiological and psychological factors. Continued evaluation of the predictive value of somatotypes and measures of muscle strength and power to evaluate and improve fencing performance overall, and the lunge component more specifically, using multivariate analysis should be pursued in future studies.

#### 4.1.3 Asymmetry

Fencing is clearly an asymmetric sports event based on its kinematic and kinetics [3, 15, 18]. Intuitively, the asymmetric sports contributed to asymmetry anthropometry due to the unilateral nature of the training. Irurtia et al. and Turner et al. [26, 53] showed that fencers had significantly higher handgrip strength and greater isokinetic leg strength on the dominant side than on the non-dominant side. Margonato and colleagues [23] conducted surveys on national-level fencers and discovered that they had higher muscle cross-sectional area in the dominant forearm. Arm and leg asymmetries were also observed in young fencers [49]

However, the effects of laterality on measures of muscle structure and performance have not been always consistently reported. Poulis et al. [48] failing to identify differences in peak knee and ankle isokinetic torques between the dominant and nondominant lower limb. It was argued that fencing movements do not necessarily induce unequal muscle growth between two sides of the lower limb. In fact, both trailing and leading legs contributed significantly to the progression of various fencing actions, especially for advance and fleche [15, 24]. Besides, Akpinar et al. [38] investigated the differences in movement accuracy, speed, multi-joint coordination, and handedness between fencers and non-fencers in a series of hand reaching tasks. They showed that fencers may have performance symmetry superior to non-fencers due to an underlying high skill in bilateral control, while Poulis also advocated that elite fencers have all-round development on their neuromuscular system [48]. In fact, the risk of injury associated with asymmetry is recognized, such that balance or weight training is often introduced to tackle with possible asymmetry-induced injuries [54–56] [36, 57]. The variation in outcome measures and training method may contribute to the inconsistency of results. Therefore, the development and role of asymmetry in fencing should be subject of future studies.

### 4.2 Extrinsic factors

#### 4.2.1 Weapon

Foil, saber, and epee are the three major weapons used in fencing sports. Each weapon has its own rules and strategies. Though the basic offensive and defensive techniques are universally applicable in the three weapons, their biomechanics may differ due to the variances in blade type (e.g. length and weight), valid target (e.g. torso with and without the extremities) and scoring technique (e.g. thrusting and cutting). Both epee and foil are thrusting weapons which score only by landing their tips on the valid area, while epee is heavier (775g VS 350-500g, same in blade length: 90cm) than foil. In spite of the limited evidence from existing studies, there was a trend in the numbers present that foil fencers had slightly higher peak velocity in mass center (1.92m/s VS 1.72m/s), weapon (2.91m/s VS 2.49m/s), and the front foot (4.56m/s VS 4.10m/s) than epee fencers during the execution of simple lunge attack [16, 18, 24, 25, 29, 33, 40–42]. Though the comparison is based on different samples, there are also signs from some performance analyses that the length of action and break was shorter in foil than in epee (5.2s VS 12.7s, 15.6s VS 18.2s) [14]. Ratio of action to break was lower in foil (1:3) than in epee (1:1.0–1.4) [1, 58], indicating quicker-finished bouts in foil. Saber (weight: 500g, length: 88cm in the blade) is most distinctive from the other two weapons by its ‘cutting’ rule. Data from research on saber biomechanics was rare, but saber was thought to have a fast tempo and a quicker burst of speed than the other two weapons [59]. Length of action/break (2.5±0.6s, 16.5±2.7s) and ratio of action to break (1:6.5) was the lowest in saber [14]. Up to date, fencing weapons were usually studies in simple movement and controlled in-lab conditions, e.g. lunge and fleche. However, in-game combat is more complex involving many extensions of the fundamental actions. Saber fencing may be even faster paced than reports.

In addition to having to bear the weight of the long weapon itself, fencers’ wrist further sustains abrupt and violent loading during the series of attack and defense fencing actions. Therefore, a well-designed weapon handle is important for reducing abnormal wrist joint motion and lowering the risk of wrist injury. Three types of handles are generally used in fencing: the French, the pistol, and the Italian handle. The French and Italian handles have evolved from the classical rapier handle from the Baroque period, with the French handle slightly contoured to the curve of the hand. The pistol handle is much like that of a pistol and commonly known as the “anatomical” or “orthopaedic” grip. Use of the pistol handle is advocated as it elicits lower activation in the adductor pollicis and extensor carpi radialis muscles compared to the French and Italian handles [22]. Moreover, the Visconti style pistol handle also promotes a more balanced activation of forearm muscles [21, 22] which would delay muscle fatigue and improve the capacity of the wrist to resists excessive motion when external forces are applied. This protective function of the pistol handle can be enhanced by placing the handle at an angle of 18°-21°, which also improves hit rate and accuracy while delaying the onset of early fatigue [21].

#### 4.2.2 Footwear and the fencing piste

Fencers’ feet are repeatedly exposed to large transient impact shock, especially during sudden forward thrusts, increasing the risk of lower limb injuries [60]. The metal carpet piste is the main source of these high impact forces. Although different overlay materials have been used for shock absorption, Greenhalgh et al. [27] could not confirm a significant attenuation of impact magnitude for different overlays.

Fencing shoes compound this problem by providing little intrinsic shock absorption, compared to standard court shoes. Geil [20] and Sinclair et al. [7] confirmed the lower shock absorption capacity of fencing shoes compared to squash and running shoes. Geil [20] did discuss the trade-off between increased shock absorption of shoes and a slower and less reliable performance, with shock absorbing materials reducing sensory information from the floor which provides important proprioceptive input for agility and balance [61]. Therefore, finding a balance between shock absorption and performance remains an issue to be resolved.

#### 4.2.3 Training and conditioning

The success of fencing was largely determined by speed and explosive strength [53]. Therefore, ballistic training is recommended to increase the rate of muscle force generation [62], with most of the improvements occurring within the first 200 to 300 milliseconds of a single lunge movement [63, 64]. Research has shown that pure fencing training regime did not induce growth of muscle strength that overrode the progression of puberty for the adolescent fencers (compared to the inactive children: increases in arm cross sectional area: 17.1% VS 6.97%, increases in grip strength: 25.81% VS 18.07%) [51]. The increases of leg muscle cross sectional area (32±7%) and body mass (16±3%) in fencers was also insignificant when the effects of body height were ruled out. The author thus recommended strength training for young fencers to complement their training routine.

Training of balance and coordination is also a fundamental element [65], By taking specific balance training, fencers demonstrated better coordination (42.36% improvement in balance score) and less body sway (7.02% less in dispersion of the center of plantar pressure) in single-leg standing tasks. When coordinating touché and lunge movements, elite fencers produced higher sword velocity (2.90±0.30m/s VS 2.52±0.29m/s) and body center velocity (0.41±0.20m/s VS 0.04±0.10m/s), compared to novice fencers [36, 40]. In their review of training programs, Turner et al. [53] reported that most programs are customized to a fencer’s ability, sex and age by coaches, based on their experience or anecdotal knowledge rather than on evidence. Turner et al. advocated the need to develop evidence-based training protocols which would consider a fencer’s biomechanics, as well as physiological and psychological factors.

### 4.3 Biomechanics of fencing

Fencing is a highly asymmetric sport, with the armed side of the body leading movement over a substantial duration of a competitive bout, and during training. Moreover, the upper and lower extremities present distinctive motion patterns, which imposes a considerable burden on the neuromuscular system, including effects of dominance on kinematics and kinetics [66]. The advance, retreat, fleche, and lunge movements, commonly used in fencing, have been evaluated using motion capture, demonstrating the greater joint motion and force output required to perform the fleche and lunge movements [31, 67].

#### 4.3.1 Posture and kinematics

In lunging, power during the propulsion phase is provided by the ankle plantarflexors and knee/hip extensors of the trailing leg, with additional contribution from the hip flexors and knee extensors of the leading leg during the subsequent flight phase [15]. Contrarily in fleche, both lower limbs provided power in a cyclical sprint-like manner. During the initial phase, the trailing leg provides the thrusting power as its ankle plantarflexors and knee extensors control the velocity of flexion at the ankle and knee joints of the leading leg. Once the trailing leg passes in front of the leading leg, the thrust-absorption cycle is repeated with a reversal of the power and absorption roles of the lower limbs [15]. Lower limb coordination and balance also significantly influence performance, with elite fencers generating greater hip flexion force of the leading leg at the end of a lunging movement and, hence, a higher sword velocity [16, 25]. Range of motion of the knee and peak hip flexion range of the trailing leg and hip flexion range of the leading leg were identified as significant predictors of lunge performance, allowing fencers to assume a low on-guard position and adjust movement of the leading leg in lunge to improve their performance [16]. Though these studies of fencing performance were based on small sample size, their subjects were mostly elite fencers who can represent the significance of fencing sports. Besides, the outcomes were quite consistent in terms of phase-contributors of lunge dynamics.

Study of in-shoe pressure revealed the asymmetric characteristics of weight bearing on the foot. Trautmann, Martinelli & Rosenbaum [3] and Geil [20] evaluated the distribution of plantar pressures during three fencing movements [3]. Load was predominantly placed on the heel of the leading foot and on the forefoot of the trailing foot during performance of a lunge and advance, with plantar pressure being a maximum under the hallux, bilaterally, during the retreat movement, regardless of the type of shoes worn. Steward and Kopetka [24] further reported time-to-peak angular velocity of the knee joint of the trailing leg and of the elbow of on the leading side to have a significant effect on overall lunge speed. The angle of the trailing foot relative to the lead foot also influenced lunge performance [68]. Gresham-Fieg, House & Zupan [32] evaluated the effects of three different angles of rear foot placement on lunge performance, confirming that placement of the rear foot perpendicular to the alignment of the forefoot produced higher magnitudes of peak and average power and average velocity of lunging.

#### 4.3.2 Joint coordination and synergy

The proximal-to-distal coupling of upper and lower limb motion ensures an effective transfer of joint segmental angular velocity of the lower limb to the maximum linear velocity of the center of mass [69], a feature which differentiates elite from novice fencers [37]. Elite fencers extended the weapon arm prior to initiating front foot movement during lunge [18]. Focusing specifically on the coordination among lower limb joints, Mulloy et al. [37] identified that elite fencers exhibited greater peak horizontal sword velocity and lunge distance in comparison to novice fencers through a clearly sequential increase in angular velocity of joint extension from the hip, to the knee, to the ankle. This kinematic sequence was claimed to be the correct technique that increase fencing success in elite fencers [39]. Do and Yiou has identified a ‘refractory period’ existing between motor tasks that had negative effects on fencing performance [42]. Elite fencers were competent to inhibit the effects by closely linking ‘touche’ movement of the arm and ‘lunge’ movement of the legs in perfect timeline [41]. Technical training should take into account of the specific fencing movement pattern, with emphasis on practicing different movement components in combination rather than in separate form [40].

#### 4.3.3 Muscle coordination and synergy

The proximal-to-distal sequence was also reported for muscle activation, with activation of the anterior deltoid of the armed upper limb, with extension of the armed hand, preceding the lifting of the lead foot at the initiation of lunging in expert fencers [40]. In contrast, novice fencers exhibited a delayed onset of upper limb muscle activity, associated with shortened propulsion phase by the trailing leg resulting in an earlier “kick off” of the trailing leg in novice fencers [18, 67]. Overall, elite fencers presented more coherent muscle synergies of the upper and lower limbs, compared to novice fencers, characterized by sequential activation of shoulder/elbow extensors of the armed upper limb and hip/knee extensors of the rear lower limb, followed by activation of the forelimb during lunging, with the ability to maintain this quasi-invariant pattern of activation despite changing task requirements during a fencing bout [39, 67]. In contrast, muscle activation patterns for novice fencers were more variable with changing task demands imposed by their opponents, often leading to interruptions in their movement [29, 33]. Therefore, novice fencers may not have consolidated neuromuscular strategies for complex, multi-segmental movements [70], while elite fencers are able to finely adjust muscle activation patterns to optimize attacking (lunge) efficiency without violating the “correct” kinematic sequence of upper and lower limb motions (as the sequence mentioned in section 4.3.2: rear knee extension-front shoulder extension-front knee extension-front knee flexion) [17].

## 5. Summary and remarks

Fencing is an idiosyncratic sport, with unique patterns of asymmetrical movements and biomechanics. Although athlete-specific (intrinsic) and external factors were identified as influencing performance and, probably the risk of injury, current evidence can be considered incomplete and of “fair” quality only. However, we did identify key points that can begin to inform practice, as well as providing a direction for future research. Foremost, fencing requires explosive force and as such, evidence regarding effects of sex, anthropometry, muscle structure and neuromuscular coordination is required. Although intuitively, effects of asymmetry have been discussed and evaluated, evidence of an effect of handedness on muscle strength should be more deeply studied. Moreover, elite fencers were found to have a higher capacity for bilateral performance of hand tasks than the novice or untrained individuals. However, neuromuscular control of multi-joint movements is essential to an elite fencing performance. A unique feature of fencing is the metal carpet piste and the poor shock absorbing characteristics of the fencing shoes which increase the magnitude of impact forces and the risk of foot/ankle and knee injuries. Strategies to mitigate impact forces while optimizing performance are required.

Current evidence is limited by the narrow scope of the research and “fair” methodological quality. Foremost, due to differences in the characteristics of different weapons and, therefore, movement requirements, findings are not transferable across weapon type [14]. Information available largely addresses the lunge component, which is understandable as it is the core movement component in fencing. Although a range of measures of lunge performance has been used, ranging from sword velocity to timing of target hit, the predictive value of these different measures to overall tactical performance and injury has yet to be determined. Moreover, fencing contest regularly lasts for more than an hour, during which fencers experience rapid alternation between resting and intensive activity [5]. Therefore, muscle fatigue and psychophysical exhaustion would influence performance measures and increase overall risk for injury and poor performance outcomes [1]. Future studies will need to evaluate effects of fatigue on fencing performance.

For future direction, current training programs mainly focus on improvement of muscle strength and power, with endurance training having received relatively less attention, despite its importance to injury prevention [71, 72]. Footwear design will also need to be addressed to reduce exposure to repetitive high magnitude impacts. Numerical modeling, in combination with neurophysiological measures of proprioception and muscle activation and performance-based outcomes, could assist in identifying optimal design criteria for fencing shoes by predicting internal loading across the geometrically complex anatomy of the foot and ankle [73, 74].

## Supporting information

S1 Checklist PRISMA checklist. (PDF)
